# *Chlamydomonas* dynein-preassembly-deficient mutants exhibit characteristic ciliary responses to viscous media

**DOI:** 10.17912/micropub.biology.001149

**Published:** 2024-03-12

**Authors:** Ryosuke Yamamoto, Yu Kitamura, Takahide Kon

**Affiliations:** 1 Department of Biological Sciences, Graduate School of Science, Osaka University, Ōsaka, Japan

## Abstract

Ciliary-dynein preassembly in the cytoplasm is critical for the assembly and movement of motile cilia, organelles that function under viscous conditions. Defects in preassembly often lead to a reduction in specific types of ciliary dyneins. Here, we investigated how environmental viscosity affects the motility of preassembly-deficient cilia in the alga *Chlamydomonas.* We found that, depending on the type of ciliary dynein deficiency, each *Chlamydomonas* mutant displays a characteristic phenotype in cell propulsion. Our results highlight not only the unique function(s) of each dynein species, but also the importance of functional coordination between dyneins for ciliary motility under viscous conditions.

**Figure 1. Motility phenotypes of Chlamydomonas dynein-preassembly-deficient mutants in viscous media:**
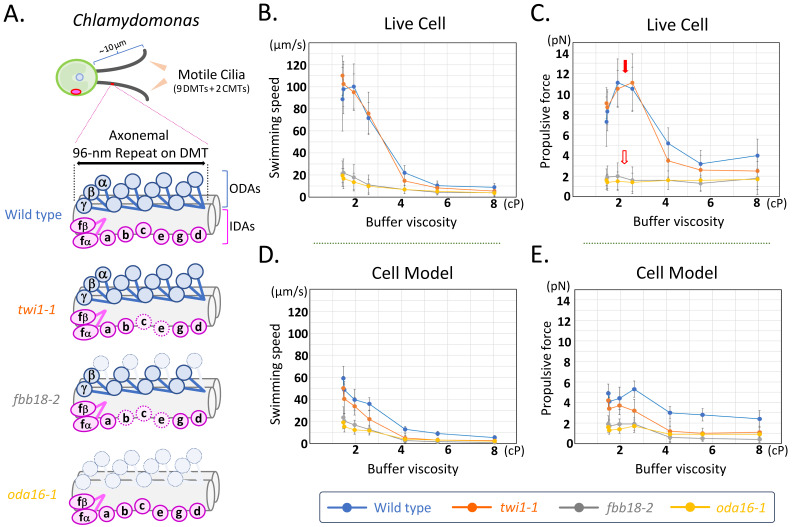
**(A)** Diagrams of ciliary-dynein composition in the *Chlamydomonas* wild-type/preassembly-deficient mutant strains used in this study. *Chlamydomonas* motile cilium (each ~ 10 µm in length) consists mainly of 9 doublet microtubules (DMTs) and 2 central microtubules (CMTs), which form the ciliary axoneme inside the motile cilium. Wild-type (blue) cells assemble all ciliary dynein species [ODA (HCs α, β and γ) and seven IDAs (IDAs a, b, c, d, e, fα/fβ and g)] on the axonemal 96-nm repeat on the DMT of motile cilia (Bui et al., 2012; Yamamoto et al., 2021). The preassembly-deficient mutant *oda16* (yellow) lacks a large amount of ODAs in cilia (Ahmed & Mitchell, 2005). The other two preassembly-deficient mutants have overlapping but distinct ciliary reduction of dyneins [ODA HCα, IDAs b, c and e for *fbb18* (gray) (Manuscript in preparation); IDAs c and e for *twi1* (orange) (Yamamoto et al., 2020)]; see also the main text for details. **(B)** Swimming speed and **(C)** ciliary propulsive force of live wild-type, *twi1* , *fbb18* and *oda16* cells (blue, orange, gray and yellow lines, respectively) under viscous conditions. Wild-type and *twi1* cells show relatively similar ciliary phenotypes in response to buffer viscosity, whereas the motility of *fbb18* and *oda16* cells was much lower than that of wild type and *twi1* . In addition, wild-type and *twi1* cells show increased ciliary propulsive force at low buffer viscosities (~ 2 - 3 cP) [red arrow in **(C)** ] compared to basal viscosity (~ 1.5 cP), whereas *fbb18* and *oda16* cells do not show such an increase in a propulsive force in response to increased environmental viscosity [blank arrow in **(C)** ]. **(D)** Swimming speed and **(E)** ciliary propulsive force of wild-type, *twi1* , *fbb18* and *oda16* cell models (blue, orange, gray and yellow lines, respectively) under viscous conditions. Reactivated models of all three preassembly-deficient mutants show reduced motility and propulsive force compared to the wild type, especially at medium to high viscosities (> ~ 5 cP), suggesting that the partial loss of ciliary dyneins in these mutants has major effects on the motility of demembranated cilia. For these swimming analyses **(B, C, D and E)** , we primarily selected cells that showed motility in the viscous conditions. From several independent measurements, we selected one measurement per strain in which the cells (n = 100 per strain) exhibited a typical swimming phenotype, and summarized the results [mean±SD] in this figure.

## Description

Ciliary dyneins are preassembled from their subunits [heavy/intermediate/light chains (HCs/ICs/LCs)] in the cytoplasm before being transported into motile cilia, organelles with many vital functions in eukaryotes (Ostrowski et al., 2011; Satir & Christensen, 2007; Satir et al., 2008; Vincensini et al., 2011) , and this "dynein preassembly" is essential for normal ciliary motility (Fowkes & Mitchell, 1998; Yamamoto et al., 2010; Yamamoto et al., 2020) . Motile cilia often function in viscous conditions in nature (e.g. mud) or in organisms (e.g. mucus/semen), and cilia must generate a strong propulsive force to function in such viscous environments where the Reynolds number becomes lower, i.e., the inertial force is recessive while the viscous force is dominant (Gray, 1930; Holwill et al., 1995) . It has been hypothesized that ciliary dyneins coordinate their activities to generate such a strong force to drive ciliary motility in these environments, and that assembly defects in ciliary dyneins result in reduced ciliary force generation and motility, ultimately causing various diseases (collectively referred to as "ciliopathies") in humans (Chodhari et al., 2004; Horani & Ferkol, 2016; Knowles et al., 2016) .

Ciliary dyneins are mainly divided into two groups: outer arm dyneins (ODAs) and inner arm dyneins (IDAs) ( **Figure 1A** ) (Gibbons, 1995; Gibbons & Rowe, 1965; Kamiya & Yagi, 2014) . In most eukaryotes, IDAs can be further subdivided into seven major species, designated "IDA a" to "IDA g" (Bui et al., 2012; Kagami & Kamiya, 1992; Yagi & Kamiya, 2012; Yamamoto et al., 2021) . Previous studies in a ciliated alga, *Chlamydomonas reinhardtii* , have shown that cilia lacking all ODAs (from the *oda1* mutant) generate a weak propulsive force compared to wild-type cilia (Minoura & Kamiya, 1995) , indicating that ODAs are the major force generator for cilia (Brokaw & Kamiya, 1987) . On the other hand, IDAs, although weaker than ODAs, also generate ciliary propulsive force. By analyzing mutants ( *ida4* and *ida9* ) that completely lack IDA c, this IDA species has been shown to be particularly important for generating high propulsive forces under medium- to high- viscous conditions (> ~ 5 cP) (Minoura & Kamiya, 1995; Yagi et al., 2005) . In addition, by comparing different mutants, each completely lacking specific dynein species, it was hypothesized that IDAs, when all normally assembled, are capable of generating a fairly constant force independent of environmental viscosity (Minoura & Kamiya, 1995) . These intriguing observations led us to investigate the ciliary phenotypes of several *Chlamydomonas* preassembly-deficient mutants that have only partial defects in ciliary dynein assembly, i.e., that retain at least some amounts of the affected dynein species in cilia, and how partial losses of ciliary dyneins affect ciliary motility under viscous conditions.

To study the ciliary phenotypes of preassembly-deficient mutants under viscous conditions compared to the wild-type *Chlamydomonas* strain (CC124), we selected the following three mutants ( **Figure 1A, see also "Reagent"** ) for both live-cell and ATP-reactivated cell-model observation: **[1]** *oda16-1* (or simply *oda16* ), which has a defect in the adapter (ODA16/DAW1) between ODAs and the intra-flagellar transport (IFT) machinery, and has a large reduction, but retains small amounts, of ODAs in cilia (80 - 90% reduction estimated by EM observation) (Ahmed et al., 2008; Ahmed & Mitchell, 2005) , **[2]** *twi1-1* (or simply *twi1* ), which has a defect in the PIH domain-containing preassembly factor TWI1, and has a selective reduction in IDAs c and e (~ 30% and ~ 20% spectral reduction, respectively) (Yamamoto et al., 2020) , and **[3]** *fbb18-2* (or simply *fbb18* ), which has a defect in the preassembly factor FBB18 and has a selective reduction in IDAs b, c and e (~ 40%, ~ 30% and ~ 15% spectral reduction, respectively), and also has a large reduction in ODA HCα (~ 90% spectral reduction) while maintaining normal levels of other ODA HCs (HCβ and HCγ) [Manuscript in preparation, for *fbb18-1* , see (Wang et al., 2022) ].

From live-cell observations and ciliary-propulsive-force calculations, we found that the live *oda16* mutant had a very low swimming speed and ciliary propulsive force compared to the wild type ( **Figures 1B and 1C** ). The response of live *oda16* cells to buffer viscosity was very similar to that of the previously analyzed *oda1* mutant, which completely lacks ODAs (Minoura & Kamiya, 1995) and generates a relatively constant force at various viscosities. This result indicates that the small amount of ODAs assembled in *oda16* cilia (Ahmed & Mitchell, 2005) is most likely not well coordinated/functional under the viscous conditions, and that the fully assembled IDAs in *oda16* cilia are capable of generating a constant force (~ 1.5 pN) independent of the surrounding viscosity. In our analysis, live *twi1* cells exhibited a similar phenotype to the wild type in viscous buffers ( **Figures 1B and 1C** ), suggesting that a combination of slight reductions in IDAs c and e ( **Figure 1A** ) does not significantly affect the ciliary response to environmental viscosity. In live *twi1* cells, we also did not observe the drastic reduction in ciliary propulsive force at medium to high viscosities (> ~ 5 cP) that was previously observed in the *ida9* mutant (Yagi et al., 2005) , which completely lacks IDA c. This result probably indicates that the slight loss of IDA c in cilia does not significantly affect the coordination of activity between different ciliary-dynein species, and/or that other dynein species can compensate for the partial loss of IDA c in *twi1* cilia.

In contrast to *twi1* but more like *oda16* , live *fbb18* cells swam very slowly compared to wild type even at the basal to low viscosities (1.5 - 3 cP) with greatly reduced propulsive force ( **Figures 1B and 1C** ), strongly suggesting that a combination of partial losses of ODA HCα, IDAs b, c and e ( **Figure 1A** ) causes miscoordination between ODA and IDA activities and/or the activities of ODAs. This result is consistent with the previous report showing that a combination of defects in both ODAs and IDAs causes very severe motility phenotypes (Kamiya et al., 1991; Piperno et al., 1990) . Interestingly, despite the relatively severe motility phenotype at low viscosity, live *fbb18* cells were able to swim at medium to high viscosities (> ~ 5 cP) like *oda16* cells ( **Figure 1B** ), with similar ciliary propulsive force generated at low viscosity (< ~ 3 cP), suggesting that the remaining dyneins in *fbb18* cilia coordinate to generate at least the basal/minimum propulsive force (~ 1.6 pN) required for cell propulsion independent of buffer viscosity. Taken together, our results from live mutant cells indicate that each mutant exhibits a characteristic phenotype in cell propulsion under viscous conditions, and that the phenotype is highly dependent on the type of ciliary dynein deficiency in each mutant.

In addition to the above results, we often observed that live wild-type cells generated more propulsive force (~ 15% increase when counting 400 cells) at low viscosities (~ 2 - 3 cP, buffer plus 2.5 - 5.0% Ficoll PM400) than at the basal viscosity (~ 1.5 cP, buffer only without Ficoll PM400), strongly suggesting that live cells are able to sense and respond to environmental viscosity for cell propulsion [as first suggested by (Minoura & Kamiya, 1995) ] ( **Figure 1C** ). Inspired by this intriguing observation, we next attempted cell-model experiments (reactivated under 1 mM ATP) ( **Figures 1D and 1E** ) to compare ciliary motility between live cells and cell models, and also to study the motility of demembranated cilia of the wild type and preassembly-deficient mutants under viscous conditions.

In the wild-type models, we found that the increase in propulsive force to the increased surrounding viscosity disappeared or was greatly attenuated ( **Figure 1E** ) [as observed in (Minoura & Kamiya, 1995) ], suggesting that this is an *in vivo* physiological response. In fact, we found that live cells cultured under medium to high (> ~ 5 cP) viscous conditions for several days often had higher motility than cells transferred to similar viscous conditions shortly before the experiments. Also, live cells cultured under the viscous conditions for several days tended to exhibit a relatively constant ciliary propulsive force regardless of the surrounding viscosity [e.g. wild-type cells cultured under viscous conditions generate around 7 - 8 pN of propulsive force at low to high viscosities (~ 1 - 6 cP)], further supporting that live cells are able to sense the surrounding viscosity and adapt to the conditions. This "adaptation" of ciliary motility to environmental viscosity may be mediated by changes in the localizations/amounts of several dynein-related proteins, including FBB18 and LIS1, which have been shown to accumulate in cilia under conditions in which ciliary motility is inhibited and/or cilia must work under high load (Austin-Tse et al., 2013; Rompolas et al., 2012) . Furthermore, we found that the motilities of both wild-type and mutant models were lower than those of live cells ( **Figures 1B and 1D** ), with all three ( *oda16* , *twi1* and *fbb18* ) mutant models showing greatly reduced motilities compared to the wild-type model at medium to high viscosities (> ~ 5 cP) ( **Figures 1D and 1E** ), suggesting that some structural and/or regulatory component(s) for high ciliary motility in live cells was lost during the demembranation step of the cell-model experiments, and/or that the concentration(s) of small molecules such as nucleotides and salts [e.g. (Omoto et al., 1996; Yagi & Kamiya, 2000) ] differed between the ciliary matrix of live cells and the reactivation buffer for the cell-model experiments. These observations also suggest that the partial loss of ciliary dyneins in the preassembly-deficient mutants has a particularly large effect on the motilities of demembranated, reactivated cilia at medium to high viscosities (> ~ 5 cP).

While testing the propulsive-force increase at low viscosities (~ 2 - 3 cP) [observed in the live wild-type cells as mentioned above ( **Figure 1C** )] in the preassembly-deficient mutants, we found that this response occurs in live *twi1* cells as in live wild-type cells, but not in the *twi1* models ( **Figures 1C and 1E** ), further supporting that it occurs only in live cells, and that a combination of a slight reduction in dyneins in *twi1* does not affect the response. In contrast, we did not observe this response in either live cells or reactivated models of *oda16* ( **Figures 1C and 1E** ), as has not been previously reported for *oda1* (Minoura & Kamiya, 1995) . This observation supports that the increase in ciliary propulsive force at low viscosities (~ 2 - 3 cP) is partly associated with ODAs, and the small amount of assembled ODAs, together with all IDAs, in *oda16* cilia ( **Figure 1A** ) is not enough to cause the increase in propulsive force. This is most likely because the remaining dyneins in *oda16* cilia are not able to coordinate their activities effectively against the increased environmental viscosity.

Interestingly, as with *oda16* , this propulsive-force increase was not observed in live *fbb18* cells ( **Figure 1C** ), which have reduced levels of ODA HCα, IDAs b, c and e ( **Figure 1A** ). We initially suspected that a reduction in ciliary ODA HCα might attenuate the response to increased environmental viscosity in live *fbb18* cells, but our brief investigation showed that live cells of *oda11* (Sakakibara et al., 1991) , a mutant lacking only the motor domain of ODA HCα, appear to retain the ability to increase ciliary propulsive force by ~ 15 - 25 % at low viscosities compared to the basal viscosity [propulsive force = ~ 5.7 - 6.2 pN (at low viscosities (~ 2 - 3 cP)) vs ~ 4.9 pN (at the basal viscosity (~ 1.5 cP)), n = 400], suggesting that a single loss of ODA HCα does not completely prevent the response. Thus, our results, together with the previous report (Minoura & Kamiya, 1995) , strongly suggest that the functional coordination between ciliary dyneins, which is impaired in both *oda16* and *fbb18* cilia, is important for live *Chlamydomonas* cells to respond to environmental viscosity, and that the partial loss of ciliary-dynein species in these preassembly-deficient mutants is sufficient to prevent this *in vivo* physiological response.

**Conclusion**

We have shown that preassembly-deficient *Chlamydomonas* mutants (both live cells and ATP-reactivated cell models) exhibit characteristic ciliary phenotypes under viscous conditions, and among them, two ( *oda16* and *fbb18* ) mutants with different dynein deficiencies are both unable to respond to increased environmental viscosity *in vivo* . Our results suggest that each dynein species has unique function(s) for ciliary motility even under viscous conditions, and also indicate that the severity of ciliary phenotypes in mutants is highly dependent on the combination of dynein species that are deficient, highlighting the importance of coordinating the activities of different ciliary dyneins. Future studies are needed to determine how the different ciliary-dynein species in eukaryotes evolved and acquired the ability to coordinate their activities to generate such a strong propulsive force of cilia, organelles that must function in highly viscous environments in nature or in organisms.

## Methods

***Cell culture***

*Chlamydomonas* cells were grown in solid or liquid TAP medium under constant light or light/dark cycles (12/12 h). Cells in liquid TAP medium were aerated as needed.

***Motility measurements***

The swimming speed of *Chlamydomonas* cells was calculated by analyzing the movies of free-swimming cells taken with the EXILIM EX-100 camera (CASIO) attached to the Olympus BX50 microscope. The calculation was performed by manually measuring the trajectory length of *Chlamydomonas* swimming using the ImageJ software (NIH). To standardize conditions, motility of both live cells and cell models was observed and measured in HMDEKP buffer [30 mM HEPES, 5 mM MgSO _4_ , 1 mM DTT, 1 mM EGTA, 50 mM potassium acetate and 1% polyethylene glycol (pH 7.4)]. We primarily measured the swimming speed of cells that showed motility in the viscous conditions.

***Cell models***

*Chlamydomonas* cell models were prepared essentially as described previously (Sakakibara & Kamiya, 1989; Yamamoto et al., 2006) . The models were reactivated under 1 mM ATP, and the motility of the models was recorded using the EXILIM EX-100 camera (CASIO) attached to the Olympus BX50 microscope. The swimming speed of the models was calculated using ImageJ as described above.

***Propulsive-force calculation***

The propulsive force generated by *Chlamydomonas* cilia was calculated as previously described (Minoura & Kamiya, 1995) using the Stokes’ equation **F = 6 π η r v** , where **η** is the viscosity of the buffer, **r** is the radius of the *Chlamydomonas* cells (assumed to be 3 μm in this study), and **v** is the swimming speed of the cells.

***Buffer-viscosity estimation***

Approximate buffer viscosity was estimated from the elution time of the buffer on a commercial 0.75-mm (diameter) viscometer (Sibata Scientific Technology, No. 026300-2). Buffer viscosity was adjusted with high-molecular-weight polymer Ficoll PM400 (Sigma-Aldrich/Cytiva).

## Reagents

**Table:** 

**STRAIN**	**GENOTYPE**	**CILIARY DEFECT**	**AVAILABLE FROM**
CC124	Wild type	N/A	*Chlamydomonas* resource center
*fbb18-2* (A9)	*fbb18*	Significantly reduced ODA HCα Reduced IDAs b, c and e (ODA HCα: ~ 90%; IDA b: ~ 40%; IDA c: ~ 30%; IDA e; ~ 15% reduction)	Upon request (Manuscript in preparation) For *fbb18-1* , see (Wang et al., 2022)
*oda11*	*oda11/dhc13*	With truncated ODA HCα Lacking the motor domain of ODA HCα	*Chlamydomonas* resource center (Sakakibara et al., 1993)
*oda16-1*	*oda16/daw1*	Reduced ODAs (all ODA HCs: 80 - 90% reduction)	*Chlamydomonas* resource center (Ahmed et al., 2008; Ahmed & Mitchell, 2005)
*twi1-1* (D4)	*twi1*	Reduced IDAs c and e (IDA c: ~ 30%; IDA e: ~ 20% reduction)	Upon request (Yamamoto et al., 2020)

## References

[R1] Ahmed Noveera T., Gao Chunlei, Lucker Ben F., Cole Douglas G., Mitchell David R. (2008). ODA16 aids axonemal outer row dynein assembly through an interaction with the intraflagellar transport machinery. The Journal of Cell Biology.

[R2] Ahmed Noveera T., Mitchell David R. (2005). ODA16p, a
*Chlamydomonas*
Flagellar Protein Needed for Dynein Assembly. Molecular Biology of the Cell.

[R3] Austin-Tse Christina, Halbritter Jan, Zariwala Maimoona A., Gilberti Renée M., Gee Heon Yung, Hellman Nathan, Pathak Narendra, Liu Yan, Panizzi Jennifer R., Patel-King Ramila S., Tritschler Douglas, Bower Raqual, O’Toole Eileen, Porath Jonathan D., Hurd Toby W., Chaki Moumita, Diaz Katrina A., Kohl Stefan, Lovric Svjetlana, Hwang Daw-Yang, Braun Daniela A., Schueler Markus, Airik Rannar, Otto Edgar A., Leigh Margaret W., Noone Peadar G., Carson Johnny L., Davis Stephanie D., Pittman Jessica E., Ferkol Thomas W., Atkinson Jeffry J., Olivier Kenneth N., Sagel Scott D., Dell Sharon D., Rosenfeld Margaret, Milla Carlos E., Loges Niki T., Omran Heymut, Porter Mary E., King Stephen M., Knowles Michael R., Drummond Iain A., Hildebrandt Friedhelm (2013). Zebrafish Ciliopathy Screen Plus Human Mutational Analysis Identifies C21orf59 and CCDC65 Defects as Causing Primary Ciliary Dyskinesia. The American Journal of Human Genetics.

[R4] Brokaw C. J., Kamiya R. (1987). Bending patterns of
*Chlamydomonas*
flagella: IV. Mutants with defects in inner and outer dynein arms indicate differences in dynein arm function. Cell Motility.

[R5] Bui Khanh Huy, Yagi Toshiki, Yamamoto Ryosuke, Kamiya Ritsu, Ishikawa Takashi (2012). Polarity and asymmetry in the arrangement of dynein and related structures in the
*Chlamydomonas*
axoneme. Journal of Cell Biology.

[R6] Chodhari R, Mitchison H.M, Meeks M (2004). Cilia, primary ciliary dyskinesia and molecular genetics. Paediatric Respiratory Reviews.

[R7] Fowkes Mary Elizabeth, Mitchell David Rees (1998). The Role of Preassembled Cytoplasmic Complexes in Assembly of Flagellar Dynein Subunits. Molecular Biology of the Cell.

[R8] Gibbons I. R. (1995). Dynein family of motor proteins: Present status and future questions. Cell Motility.

[R9] Gibbons I. R., Rowe A. J. (1965). Dynein: A Protein with Adenosine Triphosphatase Activity from Cilia. Science.

[R10] (1930). The mechanism of ciliary movement.—VI. Photographic and stroboscopic analysis of ciliary movement. Proceedings of the Royal Society of London. Series B, Containing Papers of a Biological Character.

[R11] Holwill M. E. J., Foster G. F., Hamasaki T., Satir P. (1995). Biophysical aspects and modelling of ciliary motility. Cell Motility.

[R12] Horani Amjad, Ferkol Thomas W. (2016). Primary ciliary dyskinesia and associated sensory ciliopathies. Expert Review of Respiratory Medicine.

[R13] Kagami Osamu, Kamiya Ritsu (1992). Translocation and rotation of microtubules caused by multiple species of
*Chlamydomonas*
inner-arm dynein. Journal of Cell Science.

[R14] Kamiya R, Kurimoto E, Muto E (1991). Two types of Chlamydomonas flagellar mutants missing different components of inner-arm dynein.. The Journal of cell biology.

[R15] Kamiya Ritsu, Yagi Toshiki (2014). Functional Diversity of Axonemal Dyneins as Assessed by in Vitro and in Vivo Motility Assays of
*Chlamydomonas*
Mutants. Zoological Science.

[R16] Knowles Michael R., Zariwala Maimoona, Leigh Margaret (2016). Primary Ciliary Dyskinesia. Clinics in Chest Medicine.

[R17] Minoura Itsushi, Kamiya Ritsu (1995). Strikingly different propulsive forces generated by different dynein‐deficient mutants in viscous media. Cell Motility.

[R18] Omoto C.K., Yagi T., Kurimoto E., Kamiya R. (1996). Ability of paralyzed flagella mutants ofChlamydomonas to move. Cell Motility and the Cytoskeleton.

[R19] Ostrowski L. E., Dutcher S. K., Lo C. W. (2011). Cilia and Models for Studying Structure and Function. Proceedings of the American Thoracic Society.

[R20] Piperno G, Ramanis Z, Smith E F, Sale W S (1990). Three distinct inner dynein arms in Chlamydomonas flagella: molecular composition and location in the axoneme.. The Journal of cell biology.

[R21] Rompolas Panteleimon, Patel-King Ramila S., King Stephen M. (2012). Association of Lis1 with outer arm dynein is modulated in response to alterations in flagellar motility. Molecular Biology of the Cell.

[R22] Sakakibara Hitoshi, Kamiya Ritsu (1989). Functional recombination of outer dynein arms with outer arm-missing flagellar axonemes of a
*Chlamydomonas*
mutant. Journal of Cell Science.

[R23] Sakakibara H, Mitchell D R, Kamiya R (1991). A Chlamydomonas outer arm dynein mutant missing the alpha heavy chain.. The Journal of cell biology.

[R24] Sakakibara H, Takada S, King SM, Witman GB, Kamiya R (1993). A Chlamydomonas outer arm dynein mutant with a truncated beta heavy chain. The Journal of cell biology.

[R25] Satir Peter, Christensen Søren Tvorup (2007). Overview of Structure and Function of Mammalian Cilia. Annual Review of Physiology.

[R26] Satir Peter, Mitchell David R., Jékely Gáspár (2008). Chapter 3 How Did the Cilium Evolve?. Ciliary Function in Mammalian Development.

[R27] Vincensini Laetitia, Blisnick Thierry, Bastin Philippe (2011). 1001 model organisms to study cilia and flagella. Biology of the Cell.

[R28] Wang Limei, Li Xuecheng, Liu Guang, Pan Junmin (2022). FBB18 participates in preassembly of almost all axonemal dyneins independent of R2TP complex. PLOS Genetics.

[R29] Yagi T, Kamiya R (2000). Vigorous beating of Chlamydomonas axonemes lacking central pair/radial spoke structures in the presence of salts and organic compounds.. Cell Motil Cytoskeleton.

[R30] Yagi Toshiki, Kamiya Ritsu (2012). Genetic Approaches to Axonemal Dynein Function in Chlamydomonas and Other Organisms. Dyneins.

[R31] Yagi Toshiki, Minoura Itsushi, Fujiwara Akiko, Saito Ryo, Yasunaga Takuo, Hirono Masafumi, Kamiya Ritsu (2005). An Axonemal Dynein Particularly Important for Flagellar Movement at High Viscosity. Journal of Biological Chemistry.

[R32] Yamamoto Ryosuke, Hirono Masafumi, Kamiya Ritsu (2010). Discrete PIH proteins function in the cytoplasmic preassembly of different subsets of axonemal dyneins. Journal of Cell Biology.

[R33] Yamamoto Ryosuke, Hwang Juyeon, Ishikawa Takashi, Kon Takahide, Sale Winfield S. (2021). Composition and function of ciliary inner‐dynein‐arm subunits studied in
*Chlamydomonas reinhardtii*. Cytoskeleton.

[R34] Yamamoto Ryosuke, Yagi Toshiki, Kamiya Ritsu (2006). Functional binding of inner-arm dyneins with demembranated flagella ofChlamydomonas mutants. Cell Motility and the Cytoskeleton.

[R35] Yamamoto Ryosuke, Yanagi Shiho, Nagao Masahito, Yamasaki Yuya, Tanaka Yui, Sale Winfield S., Yagi Toshiki, Kon Takahide (2020). Mutations in PIH proteins MOT48, TWI1 and PF13 define common and unique steps for preassembly of each, different ciliary dynein. PLOS Genetics.

